# Southern Alaska as a source of atmospheric mineral dust and ice-nucleating particles

**DOI:** 10.1126/sciadv.adg3708

**Published:** 2023-08-16

**Authors:** Sarah L. Barr, Bethany Wyld, James B. McQuaid, Ryan R. Neely III, Benjamin J. Murray

**Affiliations:** ^1^ School of Earth and Environment, University of Leeds, Leeds, UK.; ^2^ National Centre for Atmospheric Science, Leeds, UK.

## Abstract

Ice-nucleating particles (INPs) influence cloud radiative properties and climate; however, INP sources and concentrations are poorly constrained, particularly in high-latitude regions. Southern Alaska is a known source of high-latitude dust, but its contribution to atmospheric mineral dust and INP concentrations has not been quantified. We show that glacial dust collected in southern Alaska is an effective ice-nucleating material under conditions relevant for mixed-phase clouds and is more active than low-latitude dust because of a biological component that enhances its activity. We use dispersion modeling to show that this source contributes to the regional INP population and that the dust emitted is transported over a broad area of North America, reaching altitudes where it could cause cloud glaciation. Our results highlight the importance of quantifying emissions and ice-nucleating characteristics of high-latitude dusts and suggest that the ice-nucleating ability of emitted dust in these regions should be represented in models using different parametrizations to low-latitude dust.

## Teaser

Glacial dust from Alaska nucleates ice and is transported to regions where it could initiate ice formation in mixed-phase clouds

## INTRODUCTION

The presence of ice in clouds has a strong control on their physical and optical properties and the processes that they control such as radiative transfer and precipitation formation (*1*, *2*). Heterogeneous freezing, triggered by the presence of ice-nucleating particles (INPs), is an important pathway for ice formation in mixed-phase clouds in the atmosphere. Hence, understanding INPs and their role in the formation of ice in clouds is of crucial importance to constrain the role of clouds in the climate system (*3*–*5*).

Despite their importance, our understanding of INPs is still relatively poor, and their sources, concentrations, and seasonal variability are poorly quantified. This is particularly pronounced in high-latitude (≥50°N and ≥40°S) regions where mixed-phase clouds, which are highly sensitive to the presence of INPs, are common (*6*). Many laboratory and field measurements have focused on low and mid-latitudes where globally important sources of INPs, such as desert dust, have been identified (*7*–*10*). However, there has been less focus on INP sources in the mid- to high latitudes (45°N to 75°N), a region critical for cloud-phase feedback (*5*). As the climate warms, the amount of liquid water relative to ice in mixed-phase clouds will increase. This leads to clouds with a higher albedo, which has a cooling effect over dark surfaces such as the ocean and, hence, a negative climate feedback (*2*, *5*). While it is clear that the cloud-phase feedback is negative, there is still uncertainty over the magnitude of this feedback (*2*, *3*, *5*, *11*). The strength of the cloud-phase feedback depends on the amount of ice in clouds, so accurate modeling of the partitioning between ice and liquid water in present-day clouds, as well as in a changing climate, is key to predicting the magnitude of the cloud-phase feedback. However, ice-related processes are poorly represented in general circulation models, and the amount of ice is often overestimated (*2*, *3*, *11*). INPs relevant for the cloud-phase feedback have not been quantified, and their sources, concentration, and influence on mixed-phase clouds remain a substantial uncertainty in estimates of cloud-phase feedback (*5*).

The high latitudes are remote from the major low-latitude dust sources in Africa and Asia; hence, the concentration of low-latitude desert dust is relatively small (*12*), and there is also strong seasonal variability in aerosol concentrations relating to transport and removal mechanisms (*13*–*15*). This means that local aerosol sources can be the dominant source of mineral dust in the high latitudes (*15*, *16*). Dust emission zones in northern high latitudes have been identified in regions including Iceland, Canada, Greenland, and Alaska (*17*, *18*). While such high-latitude dust (HLD) sources account for a relatively small fraction of global dust emissions [~5% (*17*)], studies have shown that dust from sources >60°N contributes up to 27% of the total dust load in the Arctic, which is comparable to the 32% contribution of African dust (*16*, *19*) to the Arctic.

HLDs have been shown to nucleate ice under conditions relevant for mixed-phase cloud formation (*20*–*22*), suggesting that they could be an important source of INPs. However, there is considerable variability in the characteristics of these sources, and there have been few studies investigating the transport of dust and INPs from HLD sources, meaning that we cannot yet make wider conclusions regarding the contribution of HLDs to atmospheric INP populations. Tobo *et al.* (*20*) and Xi *et al.* (*22*) report the ice-nucleating activity of glacial outwash sediments, from Svalbard, Norway and the Yukon, Canada, respectively. Sediment produced by glacial processes is one of the main contributors to HLD emissions (*17*), but the physical and chemical properties are influenced by the local bedrock and environment. Dust from other origins, such as sediment derived from volcanic material in Iceland, as studied by Sanchez-Marroquin *et al.* (*21*), again has different composition and properties (*23*). Dust can also be altered by processes occurring during transport, deposition, and uplift (*18*). For example, glacial outwash sediment from Svalbard studied by Tobo *et al.* (*20*) had a biogenic component to its ice-nucleating activity, rather than being controlled purely by mineralogy, suggesting mixing or growth of biological material during transport in the fluvial environment. The physical, chemical, and biological processes that dust and sediments are exposed to and the resulting effect on the ice-nucleating properties of the material will vary depending on environmental conditions such as the local climate and land cover. Sediments studied by Tobo *et al.* (*20*) originated from largely ice- and vegetation-free areas of Svalbard, where less than 10% of the landscape is covered in vegetation and there are no tall trees. However, in many other regions, glacial sediments are transported by rivers in vegetated or forested mountain catchments. There are biological INP sources in forests in the northern high latitudes (*24*, *25*), and hence, there is potential for the ice-nucleating ability of transported sediments to be enhanced by the presence of biogenic material from abundant vegetation. There have been few studies of the ice-nucleating ability of dust originating from such regions.

**Fig. 1.**
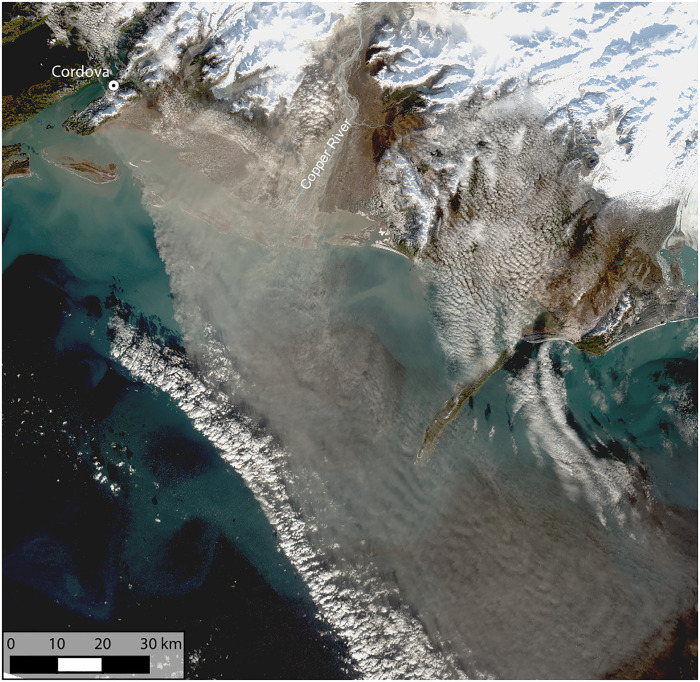
True color image of the Copper River Delta. Derived from Landsat 8 Collection 2 Tier 1 calibrated top-of-atmosphere reflectance of a dust event on 31 October 2020.

The south coast of Alaska is one of the most active sources of glacial dust in the northern high latitudes (*17*, *18*, *26*), and dust events have been observed as early as 1910 (*27*). Here, numerous glacially fed rivers originate in the Wrangell and Chugach Mountains and flow to the Gulf of Alaska, transporting glacial sediment that is deposited on floodplains at their terminus. The largest of these rivers is the Copper River, also known as Atna’tuu (Ahtna Athabascan) or Eekhéeni (Tlingit). The watershed of the Copper River spans 62,000 km^2^ of southern Alaska and encompasses ice- and snow-covered mountain terrain, boreal forests, temperate rainforests, and wetlands (fig. S1). Glaciers cover 18% of the catchment (*28*) and load the river with melt-water and sediment produced by glacial processes, resulting in around 70 million tons of sediment being transported by the river each year (*29*), the highest annual suspended-sediment load in Alaska (*30*). Some of this sediment is deposited on the Copper River Delta. In late summer or autumn, when the river levels are at their lowest and north-easterly winds down the river valley are prevalent (*26*), this fine glacial sediment is lofted into the atmosphere, resulting in large dust events that can last several days or weeks and extend hundreds of kilometers over the Gulf of Alaska, as shown in Fig. 1. Single events have been estimated to transport up to 80 kilotons (kt) of dust from the Copper River Valley (*31*), and dust from the Copper River has been shown to play an important role in the transport of minerals to the Gulf of Alaska (*31*, *32*). However, the ice-nucleating ability of the dust has not yet been quantified.

Here, we investigate the ice-nucleating ability of glacial dust from the Copper River Delta by collecting size-segregated samples of airborne dust from the Copper River Delta and characterized its ice-nucleating activity in a laboratory study. We then use particle dispersion modeling to model the transport of this dust and estimate atmospheric INP concentrations to test the hypothesis that the Copper River Valley is an important source of INPs for the North Pacific and the northern North American continent.

## RESULTS

Making measurements in high-latitude regions is often difficult because of their remoteness, lack of infrastructure, and potentially extreme environments; hence, the choice of a field site was an important consideration. As well as being an important dust source, the Copper River Delta can be accessed using a normal 4 × 4 vehicle via the Copper River Highway, a gravel road from the nearby town of Cordova (60.5°N, 145.8°W). This presented an excellent opportunity to access an active dust source region directly. We used portable battery-operated equipment that can easily be carried by one person, meaning sampling could be undertaken with minimal resources.

### Fraction frozen and INP concentration

**Fig. 2.**
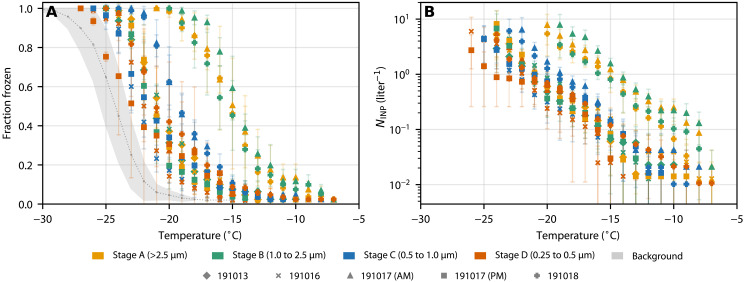
Size-resolved ice-nucleating activity of Copper River Valley dust. (**A**) Fraction frozen for all samples. (**B**) INP concentration per standard liter of air for all samples. Different colors represent each of the four collection stages, and different symbols distinguish different sampling days. The mean and SD of handling blanks shown in (A) represent the background INP activity used in calculating the error shown by error bars in (B).

We collected size-resolved samples of airborne dust during a field campaign to the Copper River in autumn of 2019. Using a multistage cascade impactor that collects samples onto substrates in four size bins (0.25 to 0.5 μm, 0.5 to 1.0 μm, 1.0 to 2.5 μm, and >2.5 μm; in pactice the upper limit to the >2.5 μm stage is around 6 μm; see Materials and Methods), we collected multiple samples during dust events, where the mass loading was 10 to 170 μg m^–^^3^ (see Table 1). Substrates from the four impactor collection stages were analyzed using droplet freezing experiments; from this, we determined fraction frozen (*f*_ice_) and INP concentrations (*N*_INP_) for each size bin. Details of each sampling period are given in Table 1 and the resulting size-resolved *f*_ice_ and *N*_INP_ values, as a function of temperature, in Fig. 2. Most of the data are above the mean handling blank, showing that the dust from this region nucleates ice at warmer temperatures than the experimental background. Both fraction frozen and *N*_INP_ show some size dependence with the larger stages, A and B, freezing at warmer temperatures. This is particularly apparent in samples 191017 (AM) and 191018, which exhibit the largest difference in fraction frozen and *N*_INP_ between stages A and B compared to C and D. This shows that larger particles contribute more to the INP population at the source. The sampling periods with higher INP concentrations [191017 (AM) and 191018] corresponded to the periods with greater dust loading (see Table 1). Other size-resolved INP concentration measurements in the North American Arctic also show that on at least some days, the INP concentrations at temperatures above ∼−20°C are greater in the supermicrometer size range compared to the submicrometer range (*33*–*35*). Hence, this may indicate that local dust sources contribute to the supermicrometer INP population across the North American Arctic.

**Table 1.** Overview of each sample including sample locations (A and B) as shown in Fig. 7. Wind speeds refer to the measured wind speed at the start of the sampling period. PM10 (the mass concentration of aerosol particles smaller than 10-μm diameter) is calculated from the mass on each stage and the sample volume. The uncertainty in PM10 values combines the SD of the mass measurements for each stage. The size bins of the stages are as follows: stage A, >2.5 μm; stage B, 1.0 to 2.5 μm; stage C, 0.5 to 1.0 μm; and stage D, 0.25 to 0.5 μm.

Date (YYMMDD)	Location	Sample volume (liter)	Wind speed (m s^−1^)	Mass per stage (μg)				PM10 (μg m^−3^)
				A	B	C	D	

191013	A	2172	13	41	16	<5	<5	26 ± 8
191016	A	1593	15	27	12	20	11	43 ± 11
191017 (AM)	A	1696	15	128	118	9	8	167 ± 6
191017 (PM)	B	1579	17	<5	10	<5	7	10 ± 7
191018	A	2255	12	40	75	27	6	66 ± 5

### Heat sensitivity of samples

We used heat tests to further investigate the INP activity of our samples and identify potential biogenic controls of the nucleation. Ice-nucleating proteins in fungus and bacteria are known to deactivate on heating to ∼100°C when immersed in water (*36*). In contrast, the mineral K-feldspar, which often controls the ice-nucleating activity of abiotic mineral dusts, does not deactivate under the standard wet-heat test conditions. Hence, we interpret a decrease in ice-nucleating activity after heating as the presence of an ice-nucleating proteinaceous biological component contributing to the activity of the sample.

**Fig. 3.**
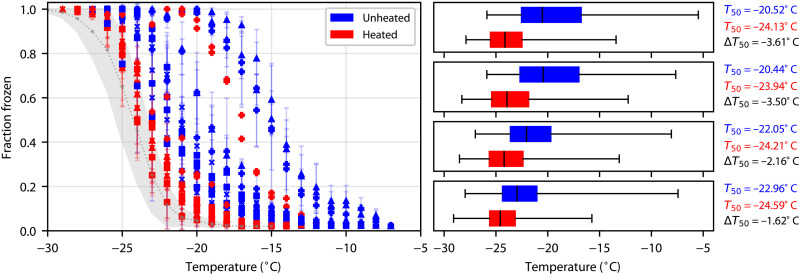
Heat tests for protein-based biological ice-nucleating entities. Fraction frozen before heating (blue) and after heating (red) of each sample along with box plots for each stage. The box plots include all of the samples; each box represents the 25th and 75th percentiles, and the whiskers cover the full spread of the data. The median freezing temperature, Δ*T*_50_, is shown with a black line.

Comparing our unheated and heated samples for all size bins (Fig. 3A) shows a clear reduction in activity. Generally, the larger size bins experienced a greater decrease in activity on heating. Comparing heated and unheated samples in each size range (Fig. 3B) shows that the most notable deactivation occurs in the larger size bins and change in the median freezing temperature, Δ*T*_50_, decreases with each size bin. This deactivation, as well as the fact that the activity was also observed to be size dependent (Fig. 2), suggests that either larger biological particles are present in the glacial dust or that large dust particles have ice-nucleating proteins attached to them. For stages A and B on 18 October, we saw a much smaller decrease in activity (2°C versus a mean of 3.6°C). This perhaps indicates the presence of either heat-insensitive mineral dust (unlikely given the analysis presented below) or heat-insensitive biological materials, such as polysaccharides from pollen. Changing weather and environmental conditions, such as soil moisture, may also affect the emissions of dust. During the field campaign, we experienced rain on 12 and 13 October followed by dry conditions. This meant that throughout the field campaign, the surface was drying; however, the soil moisture across the delta is likely to vary, and the drying itself would depend on sediment composition, particle size, and location. Hence, it is possible that the characteristics of the dust emissions, such as the size distribution and biological content, change as this drying process progresses and dust can be emitted from different areas. This suggests that the environmental conditions in the watershed influence the ice-nucleating activity of HLDs.

### Ice-active site density

**Fig. 4.**
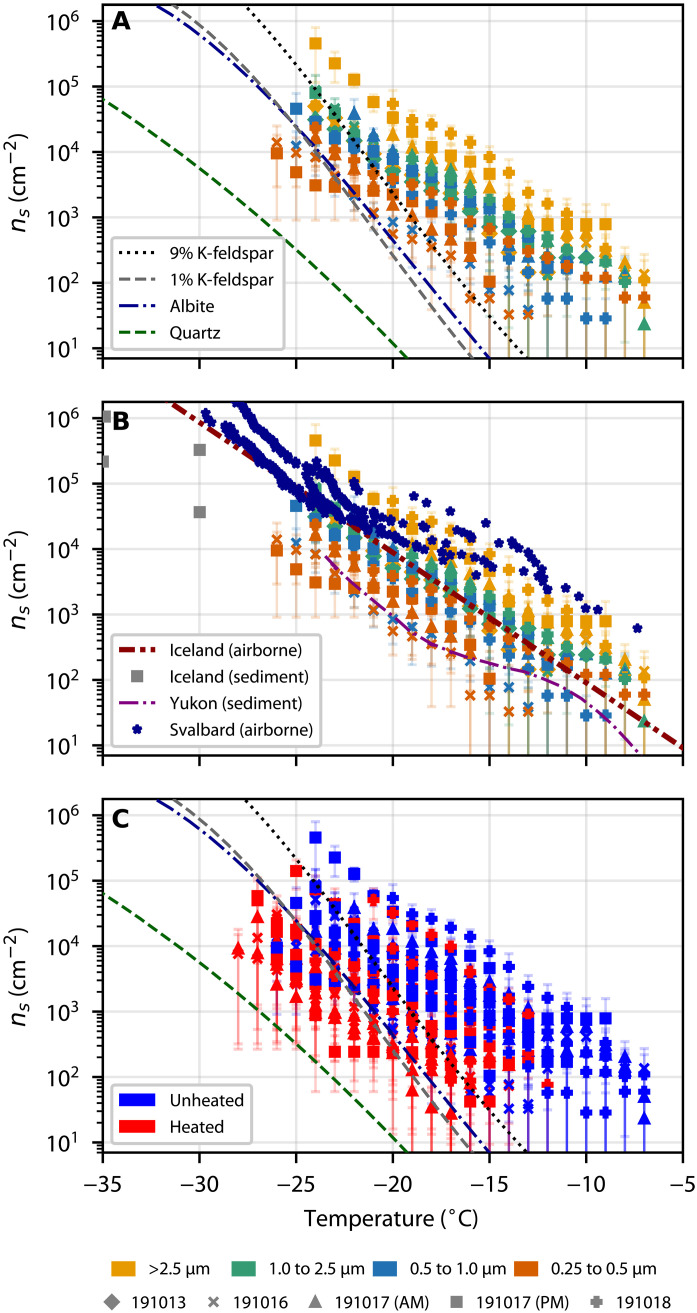
Size-resolved active site density (*n_s_*) of Copper River samples. (**A**) Comparison of *n_s_* for Copper River dust to *n_s_* parametrizations of ice-active minerals from the work of Harrison *et al.* (*39*) scaled to the mineral content of our bulk sample (from XRD analysis): 9% K-feldspar, 15% quartz, and 58% albite. (**B**) Comparison of *n_s_* for Copper River dust to airborne dust samples from Iceland (*52*) and Svalbard (*48*) and sediment samples from Iceland (*42*) and the Yukon (*22*). (**C**) Comparison of the *n_s_* for Copper River dust before and after heating, as well as to the same minerals as in (A).

To quantify the ice-nucleating activity of our Copper River dust samples, we normalized the INP concentration data to the aerosol surface area derived gravimetrically to give the ice-active site density, *n*_*s*_ (Fig. 4). The general trend across the five samples (that we could obtain gravimetric data for) was that the larger particles had a greater *n*_*s*_ than the smaller ones, by approximately one order of magnitude. Reicher *et al.* (*37*) reported that desert aerosol particles in Israel of *D*_50_ 3.2 μm had a slightly larger *n*_*s*_ than 1.0-μm particles. Similarly, in Leeds, UK, Porter *et al.* (*38*) report that the 2.5- to 10-μm size range had a greater *n*_*s*_ than the <2.5 μm. However, there are other locations where the activity of aerosol does not increase with particle size; for example, a measurement from Svalbard indicates that particles between 0.5 and 10 μm had very similar *n*_*s*_ values. Overall, it seems that for Copper River Valley dust, the larger particles have a greater ice-nucleating activity (on a per surface area basis) than the smaller ones. Because accumulation mode particles will have a longer lifetime than the coarse mode in the atmosphere, we would therefore expect the overall activity of Copper River Valley dust to decrease on transport.

X-ray diffraction (XRD) of our bulk surface samples (sieved to 45 μm) highlights the presence of a number of known ice-active minerals, namely, potassium (K-) feldspar (8.94%), quartz (15.41%), and albite (57.61%; as well as 5.86% muscovite, 7.99% calcite, and 4.19% other). Hence, in Fig. 4A, we plot the corresponding scaled *n*_*s*_ curves for the three minerals with the greatest ice-nucleating ability (K-feldspar, quartz, and albite) (*39*). These proportions were derived for 45-μm sieved dust, and the amount of these minerals in the aerosol fractions is very likely different (most likely smaller); hence, quantitative comparison with our measured *n*_*s*_ values should bear this in mind. Nevertheless, we can draw useful conclusions from this comparison. Because of its high ice-nucleating activity and abundance, we would expect K-feldspar to be the most important mineral for ice nucleation. However, our samples show a much shallower slope and higher activity at warmer temperatures (Fig. 4A) compared to a scaled parametrization of pure K-feldspar. Similarly, both albite and quartz are considerably less active than our samples. This suggests that conversely to low-latitude African desert dust (*40*, *41*), the activity of dust from the Copper River is not controlled by K-feldspar or other ice-active minerals at temperatures above about −20°C. At temperatures below −20°C, K-feldspar may account for an increasing proportion of the ice-nucleating activity of our samples.

In Fig. 4B, we compare our *n*_*s*_ values for Copper River Valley dust to *n*_*s*_ for other northern high-latitude samples. Comparing our Copper River Valley results to *n*_*s*_ for Icelandic dust sampled from an aircraft reveals that the dust from the two very different locations has very similar activity and the parametrization of the Icelandic dust fits our data well. Xi *et al.* (*22*) report the ice-nucleating activity of dust samples from a glacial valley in the Yukon, Canada (on the other side, Chugach mountain range, 300 km away from the Copper River Delta). Their results fall at the low end of the range of our data. Porter *et al.* (*38*) report size-resolved *n*_*s*_ values for a sample collected from a ship near Longyearbyen in Svalbard. As mentioned above, they do not observe a clear dependence on aerosol particle size, but their *n*_*s*_ values do overlap with the upper end of our range of *n*_*s*_ values. Paramonov *et al.* (*42*) used a continuous flow diffusion chamber to study the ice-nucleating activity of Icelandic dust (among others); their *n*_*s*_ values extend our literature comparison to lower temperatures. They found that *n*_*s*_ for 200-nm particles was greater than that for 400-nm particles, which is the opposite trend to what we observed. This comparison with the literature suggests that the ice-nucleating activity of aerosol around the Arctic varies by at least two orders of magnitude and that the size dependence of *n*_*s*_ also varies.

In Fig. 4C, we plot the *n*_*s*_ values for unheated and heated samples of Copper River Valley dust (this is the same data as in Fig. 3C). The bulk of the samples are heat sensitive across the whole temperature range and fall into the regime defined by the minerals after heating. Note that the mineral lines are defined using mineral proportions determined by XRD for a 45-μm sieved fraction, and the fact that the heated *n*_*s*_ values fall below the K-feldspar line indicates that there is less K-feldspar in the sample than the 8.94% defined by XRD. Xi *et al.* (*22*) report that the ice-nucleating activity of dust samples from a glacial valley in the Yukon, Canada, was dominated by biological material above −15°C. They did a further test using ammonium salts, which are known to enhance the ice-nucleating activity of minerals (*43*), to show that the INP population below −15°C was dominated by mineral particles. This contrasts with dust from the Copper River Valley where there is heat sensitivity to below −25°C. This is consistent with Fig. 4B where we find that the Copper River valley dust is as much as two orders of magnitude more active than that from the Yukon. This difference is perhaps related to the watershed and vegetation cover at the two sites. Xi *et al.* (*22*) sampled dust originating in the Äay Chù Valley and the Kaskawulsh glacier. The Äay Chù River begins at the Kaskawulsh glacier and runs approximately 25 km before terminating in Kluane Lake; this is in contrast to the Copper River, which is over 450 km long and has a watershed of over 62,000 km^2^, much of which is vegetated. The smaller watershed means that there is less vegetation and less variability in vegetation types [determined by comparing the land cover of the Copper River watershed (fig. S1) with satellite imagery and leaf area index of the Äay Chù Valley (from NASA Worldview)]. Vegetation is related to the various fungal and bacterial entities that are known INPs. In addition, ice-nucleating proteins can become bound to mineral particles when suspended in water (*44*); this may occur in river water leading to ice-active proteins being bound to mineral particles. Hence, the greater biological INP content of the Copper River Valley dust compared to that of the Yukon samples may be evidence that the ecosystem of the watershed defines the ice-nucleating ability of the dust that is emitted.

### Dust transport from the Copper River Valley

To assess the contribution of the Copper River Valley to INP concentrations across the wider region, we used the FLEXible PARTicle dispersion model (FLEXPART) to run 10-day forward trajectories of particles released from the Copper River Delta. FLEXPART is a particle dispersion model used to simulate the transport of air parcels in the atmosphere. Tracers are given properties that represent mineral dust, and removal processes, such as scavenging by rain and snow, are included (see Materials and Methods for details).

**Fig. 5.**
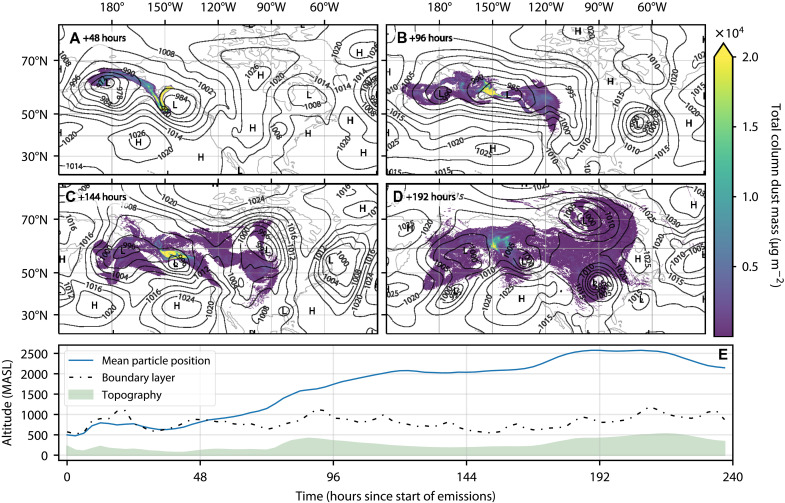
FLEXPART results from a 10-day simulation of a dust event at the Copper River Delta. (**A** to **D**) Transport of 15 kt of dust that was released over 4 days, starting 14 October 2019. The total column dust mass [0 to 10,000 meters above ground level (magl)] for 48, 96, 144, and 192 hours after the start of the emission period is shown. (**E**) Vertical profile of the mean position of released particles (blue line), the boundary layer height (dashed line), and topography (green) along this trajectory.

In Fig. 5, we show the results of one simulation where 15 kt of dust was released over 4 days, starting on 14 October 2019. Emissions from the Copper River are controlled by strong winds from the north or north east. These winds arise because of a pressure gradient driven by high-pressure systems over Alaska and a low-pressure system over the Gulf of Alaska (*31*, *32*). On a smaller scale, wind speed and direction are influenced by local topography; air is channeled down river valleys and into the Gulf of Alaska, leading to “gap” winds (*45*). We observed this during our sampling period where wind speeds were very low (<2 m s^−1^) between Cordova and the western edge of the river delta and then rapidly increased, up to as much as 25 m s^−1^, once on the delta itself. These gap winds lead to initial emissions from the Copper River being transported southward, as can be seen in our model results with a plume of high total dust mass extending over the Gulf of Alaska.

After 48 hours, the observed pressure gradient is reduced, and the dust plume is influenced by two low-pressure systems: the first to the west of the Copper River and another to the south, over the Gulf of Alaska. After 48 hours, we observe two “arms” in the modeled dust plume where dust has been entrained in both of these systems. Low-pressure systems such as those observed during this period are common in this region; in particular, the Aleutian low is a semipermanent low-pressure system located near the Aleutian Islands (to the southwest of the Copper River Delta). The position of this system has been shown to play an important role in controlling dust emissions and transport from the southern coast of Alaska (*32*). After 96 hours, the modeled dust has been transported over the Bering Sea and western North America. While the total dust mass is relatively low in some regions, a region of high concentration (0.02 g m^−2^) remains close to the southern coast of Alaska. This pattern persists after 144 hours and, after both 96 and 144 hours, is associated with the center of a low-pressure system. This suggests that the circulation prevents transport over a larger area and further reinforces that the position of the Aleutian low and other low-pressure systems in the Gulf of Alaska have a strong control on the transport of dust from the Copper River. After 192 hours, the low-pressure system begins to dissipate, and the dust begins to disperse. However, total dust mass loading of around 0.002 g m^−2^ is predicted. These mass loadings are of a similar magnitude to those predicted for dust in this region transported from lower-latitude sources (*16*).

The vertical profile in Fig. 5 (bottom) shows the mean trajectory of emitted particles. For the initial 48 hours of the simulation, much of the dust remains within the boundary layer; however, after 48 hours, this trajectory begins to increase in altitude; this coincides with the interaction with low-pressure systems, suggesting that entertainment in these systems may contribute to the vertical transport of dust out of the boundary layer. The vertical trajectory reaches altitudes of 2500 m above sea level (MASL) and shows that much of the emitted dust has been transported out of the boundary layer, meaning there is potential for this dust to reach regions in the atmosphere where temperatures are low enough for the dust to nucleate ice and influence clouds. To investigate this further, we combined modeled dust concentrations with our parametrization of *n*_*s*_ for dust from the Copper River and ambient temperatures [from ERA5 reanalysis data (*46*)] to estimate atmospheric INP concentrations.

**Fig. 6.**
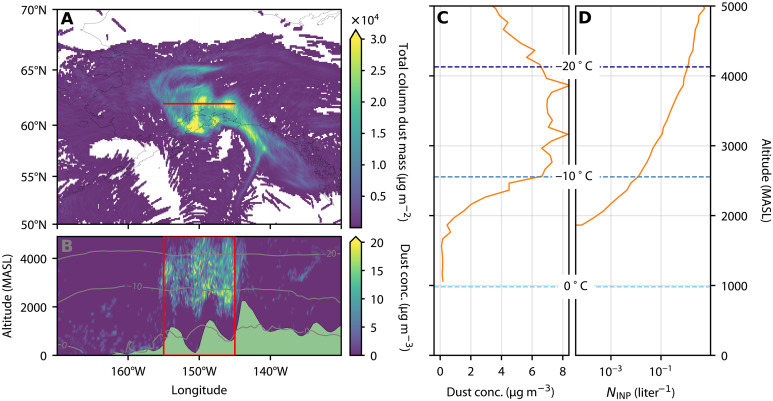
Case study of dust and INP concentrations after 60 hours. (**A**) Total dust mass integrated over a 0- to 5000-m column. (**B**) Vertical transect of dust concentrations along the red line shown in (A). Isotherms (from ERA5 reanalysis data) are shown in gray, and the topography (from FLEXPART output) is shown in green. (**C**) Vertical profile of mean dust concentration calculated within the red box shown in (B). Dashed lines represent isotherms of mean temperature in the same region. (**D**) Vertical profile of ambient INP concentration calculated using our *n*_*s*_ parametrization, dust concentrations shown in (C), and the mean temperature.

Figure 6B shows dust concentrations between 0 and 5000 MASL at 100-m vertical resolution along a transect at 62°N and, in agreement with Fig. 5, highlights vertical transport of dust with concentrations exceeding 15 μg m^−3^ up to 5000 MASL. We calculated mean dust concentrations in this region of high concentrations (within the red box), shown in Fig. 6C. It is clear from Fig. 6 (B and C) that the peak in dust concentrations is between approximately 2000 and 4000 MASL, where the temperatures range from −5° to −20°C. The ambient INP concentration reveals appreciable INP concentrations above approximately 2000 MASL, where the temperature drops below −5°C (and is therefore within the constraints of our *n*_*s*_ parametrization). At approximately 4000 MASL, dust mass concentrations are still as high as 6 μg m^−3^ and *N*_INP_ exceeds 1 liter^−1^. This is an appreciable INP concentration and is within the range that is thought to substantially reduce supercooled water content and alter cloud radiative properties (*4*); hence, the INPs from the Copper River at the concentration that we have estimated are likely to have an impact on mixed-phase clouds in this region.

Vergara-Temprado *et al.* (*12*) estimated a global distribution of INPs based on feldspar and marine organics. Feldspar is an important component of desert dust and therefore can be used to represent the contribution of desert dust to global INP concentrations. In the region of the Copper River and Alaska, they estimate an annual mean INP concentration, based on feldspar, of approximately 1 × 10^−4^ liters^−1^ at −15°C. This is considerably lower than the concentrations that we have modeled in this study at −15°C, suggesting that INPs originating from the Copper River could dominate over those from low-latitude sources.

There are some limitations with the simple modeling approach used in this study, namely, that we used an estimation of emitted dust rather than modeling it with a dedicated emission scheme. To investigate the effect of varying the initial mass of dust emitted, we completed the same analysis with total dust emissions between 1 and 80 kt. These encompass the range of total mass emissions estimated from different methods during a large dust event (*26*) but could also represent dust events of different sizes or intensities. We found that the INP concentrations at −20°C ranged from 0.05 to 10 liter^−1^ (fig. S4); this shows that even with a more conservative estimate of the total dust emissions, INP concentrations still exceeded 0.1 liter^−1^. In addition, in this study, we have investigated emissions from one source; however, the south coast of Alaska has many similar regions where dust emissions have been observed concurrently. During such events, the total mass emitted across numerous active dust sources is likely to be considerably higher than our estimates, and hence, atmospheric dust and INP concentrations could also be much higher. With this in mind, our calculations not only represent a reasonable first estimate of INP concentrations but also highlight the need for further study and better representation of emissions from HLD sources in global models.

## DISCUSSION

In this study, we show that glacial dust from the Copper River, Alaska, nucleates ice at temperatures relevant for mixed-phase clouds. Using FLEXPART for particle dispersion modeling, we show that dust from the Copper River may contribute to aerosol concentrations over a large geographical area. In combination with the size-resolved ice-nucleating activity measurements, modeling shows that this dust can be lofted by meteorological phenomena typical of this region, to altitudes where it is sufficiently cold that it can contribute a substantial INP population that out-competes INPs associated with distant desert sources. The Copper River Valley is one of many such sources on the south coast of Alaska and the wider Arctic; hence, these results indicate the importance of the inclusion of HLD in global models.

Using XRD analysis, we identified the mineral composition of this dust and found minerals that are known to be important for ice nucleation, such as potassium feldspar. We were able to compare our results to parametrizations of these ice-active minerals and found that the parametrizations did not match the activity of our samples, suggesting that the observed ice-nucleating activity was not controlled by the mineral composition of the particles, in contrast to dust from low-latitude deserts. This was further supported by heat testing the samples, which revealed that all samples were sensitive to heating, with the most deactivation observed in the larger size bins. From these results, we conclude that there is a heat-sensitive biogenic component that controls the nucleation, particularly at temperatures warmer than −20°C, and propose that this could be a result of the mixing of glacial dust with biogenic material during transport or growth of biogenic material over time. This finding is of particular importance because it shows that not only do dust concentrations need to be correctly modeled but also parametrization of INPs must be adapted to correctly represent high-latitude sources to better represent primary ice production and its role in cloud properties and climate feedbacks. In addition, we found that our samples differed from those from other similar high-latitude sources, such as glacial dust studied by Xi *et al.* (*22*), and were considerably more active (higher *n*_*s*_ values), which may be due to greater biogenic content in our samples. This suggests that the local environment, specifically the river catchment, influences the processing of glacial dust and alters the ice-nucleating properties of transported sediments. This is also an important consideration when representing HLD sources in global models, but further studies are needed to better understand the variability in ice-nucleating activity of HLD.

We have investigated one important HLD source; however, there are still very few studies of this nature, and there are many dust emission regions in northern and southern high latitudes that have not been studied. Linking field observations with modeling of dust transport is a crucial step in determining the contribution of HLD to atmospheric INP concentrations; however, many previous studies have focused on either observations or modeling. For example, Tobo *et al.* (*20*) and Xi *et al.* (*22*) identified high-latitude sources of INPs but did not investigate the transport and atmospheric concentration of INPs from these sources, whereas Shi *et al.* (*16*) and Kawai *et al.* (*47*) studied the contribution of HLDs to INPs in the Arctic but used a single parametrization to represent all HLDs. In addition, studies such as those of Sanchez-Marroquin *et al.* (*21*) and Kawai *et al.* (*47*) use global aerosol models that require substantial resources and expertise to run. The approach outlined here offers a methodology to investigate potential INP sources, from observations at the source to modeling atmospheric concentrations, that could easily be applied to other HLD sources. Using portable battery-powered sampling equipment, which is easy to carry and deploy in the field, opens up the possibility of making measurements in more inaccessible regions where larger-field campaigns would be impractical and expensive. In addition, we chose to use FLEXPART over a more complex aerosol model because it is a computationally inexpensive, open-source model that can be easily tailored to different scenarios and run without extensive modeling experience. Applying this approach to other HLD sources would provide a comprehensive first estimate of INP concentrations, which can be used to help inform which dust sources need to be included in global models.

## MATERIALS AND METHODS

### Sampling location and field campaign

**Fig. 7.**
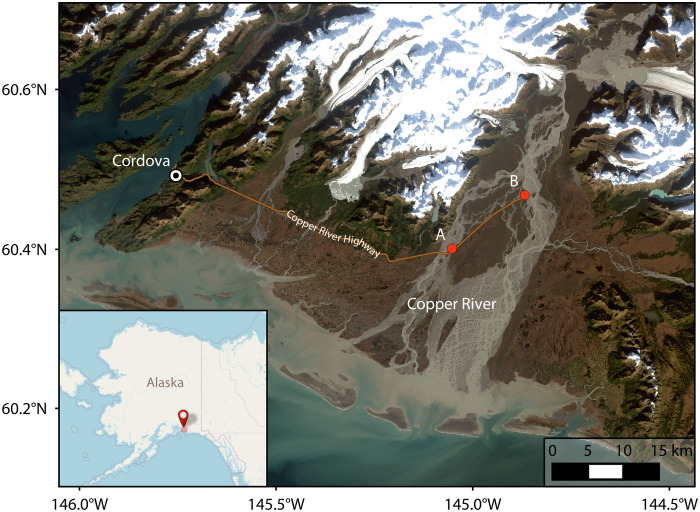
The Copper River Delta showing sampling locations A and B, Cordova, and the Copper River Highway. Background image derived from Landsat 8 Collection 2 Tier 1 calibrated top-of-atmosphere reflectance.

Sampling was conducted during a field campaign between 11 and 21 October 2019. The Copper River Highway was used to access the western side of the delta where sampling locations were chosen in regions with visible dust emissions (Fig. 7). During the 10-day period of the field campaign, dust events (i.e., days when dust emissions were observed from the surface and airborne dust was visible over a wide area) occurred for 8 days.

### Sample collection

#### Airborne dust sampling

**Fig. 8.**
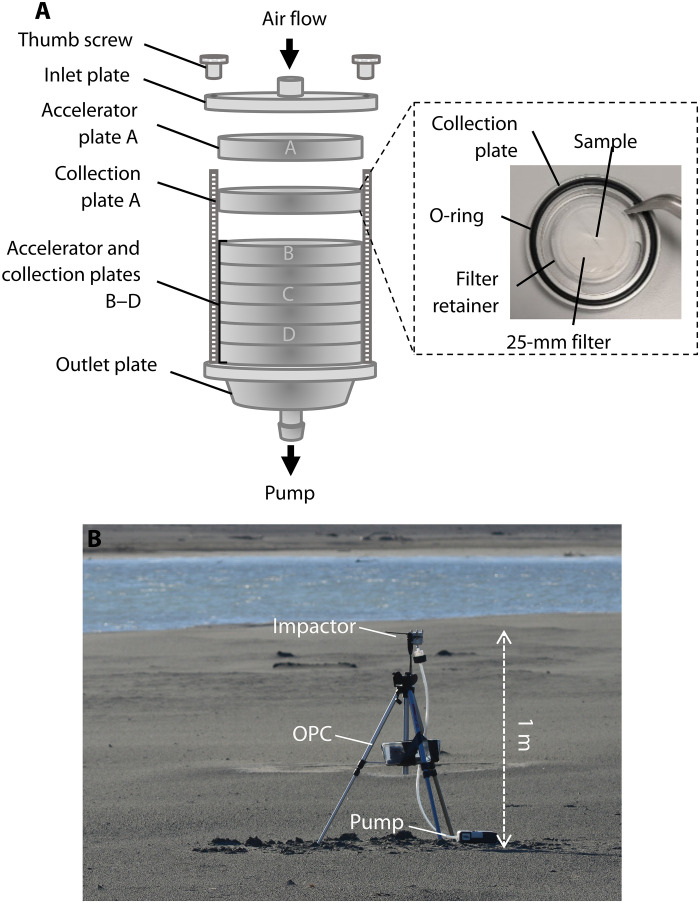
Size-resolved dust sampling in the Copper River Valley. (**A**) Sioutas Personal Cascade Impactor. Sampled air passes through accelerator plates A to D in turn and particles above the cut-off size for each plate (A, >2.5 μm; B, 1.0 μm; C, 0.5 μm; and D, 0.25 μm) are collected on to the corresponding collection plate. Collection plates (inset) consist of a 25-mm collection substrate, filter retainer, and nitrile O-ring to maintain an airtight seal. (**B**) Cascade impactor and optical particle counter deployed in the Copper River Valley on a tripod.

Airborne dust was sampled using a multistage cascade impactor (Sioutas Personal Cascade Impactor; SKC Ltd., UK), as shown in Fig. 8A, which collects size-resolved aerosol samples onto thin substrates for offline analysis. The impactor consists of collection stages A to D, which leads to aerosol being sorted into four size bins: 0.25 to 0.5 μm, 0.5 to 1.0 μm, 1.0 to 2.5 μm, and >2.5 μm. A flow rate of 9 liters^−1^ is required, which was provided by a battery-powered pump (Leland Legacy Pump; SKC Ltd.). Filters (diameter, 25 mm; Nuclepore track-etched membrane polycarbonate filters; Whatman, UK), with 0.05-μm pore size, were used as impactor substrates on each of the four impactor collection stages. An optical particle counter (OPC-N2; Alphasense, UK) was used alongside the cascade impactor to provide binned particle size distributions, and both the OPC and impactor were mounted on a tripod at a height of 1 m above the surface (Fig. 8B). During the measurement campaign, the OPC was unable to capture the high dust concentrations and eventually failed, most likely because of the optics becoming obscured/blocked by dust; therefore, an alternative gravimetric approach to determine the amount of aerosol sample was used. Wind speed was measured at hourly intervals during each sampling period using a portable three-wind cup anemometer (Skywatch Eole; JDC Electronic SA, Switzerland). Measurements were taken at the location of the sampler with the anemometer handheld above the observer’s head and so approximately 2 m above the surface, compared to the 1-m height of the sampler. This was to ensure that measurements were not influenced by the wind being blocked by the person holding the anemometer. The instantaneous, 30-s average, and maximum wind speeds were recorded. The results presented in Table 1 are the 30-s average wind speed at the start of the sampling period.

Sampling efficiencies of both the impactor and the OPC are affected by wind speed and direction, the combination of which results in sampling biases; this was an important consideration during our sampling period due to the high wind speeds observed. The orientation of sampling inlets in relation to the prevailing wind direction influences the sampling efficiency, and therefore, sampling biases can be minimized by careful positioning of the instruments (*48*). Hence, to achieve optimal sampling efficiency, the impactor was deployed vertically with the inlet upward (90° to the wind), and the OPC was deployed with the inlet facing into the wind (0° to the wind). The effect of wind speed on sampling efficiency becomes more pronounced as particle size increases. We modeled the particle losses at a range of wind speeds using an open-source particle loss calculator (*49*), the results of which are shown in fig. S2. We found that between 0 and 2.5 μm (stages B to D), particle losses are minimal. However, above 2.5 μm, the sampling efficiency quickly decreases, reaching 0 at around 6 μm for wind speeds of 16 m s^−1^. This implies that although stage A has no defined upper size limit, we are unlikely to have sampled particles >6 μm and that samples on stage A would be biased toward smaller sizes. This was considered in the calculation of specific surface area as outlined below.

Three sets of impactor collection stages were prepared at the University of Leeds and transported to the field site. The impactors were taken apart and cleaned with isopropyl alcohol, then rinsed with ultrapure water (CHROMASOLV water for high-performance liquid chromatography; Sigma-Aldrich), and lastly dried using dry nitrogen. Collection substrates were installed on each stage using tweezers, and then, each set of collection stages was wrapped in Parafilm and sealed in a sterile bag until ready to be used. Of the impactors prepared in Leeds, two were used for sampling and one as a handling blank, whereby no sample was collected on the collection substrates, but they were analyzed using the same protocol as the samples to assess the background INP activity. For subsequent samples, the impactors were prepared in Alaska: At the end of each sampling period, the impactor was sealed in a bag and transported from the field site to a nearby hotel; here, the substrates were removed from the impactor using tweezers, placed into prerinsed 50-ml centrifuge tubes, and stored in a freezer at −18°C. The collection stages and filter retainer were cleaned again using isopropyl alcohol and ultrapure water before being reloaded with new substrates and sealed until the next sampling period. The impactors were left to dry with a sterile bag placed over them. When removing samples and installing new substrates, the impactor was placed inside a freshly opened sterile polyethylene bag and then loosely closed around the hands of the person preparing the impactors; the entire process could then be completed inside the bag. In the absence of a laminar flow hood, we hoped that this would reduce potential contamination; while the exposed filters were still exposed to potentially unclean air from the room, they would be protected from particles falling onto them, for example, from clothing. The handling blanks prepared in Alaska following this protocol did not show a higher level of background INP activity when compared to the handling blanks prepared in Leeds. We therefore assume that there was no additional contamination when the impactors were prepared in the field as opposed to in the laboratory in Leeds.

#### Surface dust sampling for XRD

In addition to the airborne samples, dust source material was collected close to the sampling locations shown in Fig. 7. Material was collected from the surface using a stainless steel scoop and briefly stored in sterile bags. While still in Alaska, the surface samples were sieved using a 45-μm stainless steel sieve (Fisherbrand, UK), which was cleaned in advance with isopropyl alcohol and ultrapure water. Once sieved, the samples were stored in prerinsed containers (Nalgene polycarbonate jars; Thermo Scientific, UK) and frozen. The sieved samples were used to investigate the mineralogy of dust from this source using XRD. The percentage of each mineral in the sample was determined using Total Pattern Analysis Solutions analysis of Rietveld refinement of powder XRD patterns.

### INP droplet freezing assay experiments

The ice-nucleating ability of airborne samples was investigated using the University of Leeds Microlitre Nucleation by Immersed Particle Instrument (μL-NIPI) for cold-stage droplet freezing experiments, following the method outlined by Whale *et al.* (*50*). Suspensions were prepared by adding 3 ml of ultrapure water to the centrifuge tube containing each substrate and agitated using a vortex mixer for 10 min. From this suspension, 1-μl droplets were pipetted onto a hydrophobic glass slide placed on a temperature-controlled cold stage. A chamber, with a digital camera, was placed on top and then flushed with dry nitrogen to inhibit condensation and frost formation. The cold stage was cooled at a rate of 1°C min^−1^, and the freezing of droplets were recorded by the digital camera, which, combined with concurrent measurement of the temperature of the cold stage, allowed the fraction of droplets frozen at a given temperature, *f*_ice_(*T*), to be determined. The concentration of INPs per volume of sampled air, *N*_INP_, as a function of temperature could then be calculated according to Eq. 1 (*48*)

$$N_{\mathrm{INP}}(T)=-\ln[1-f_{\mathrm{ice}}(T)]\frac{V_{\mathrm{wash}}}{V_{\mathrm{droplet}}V_{\mathrm{air}}}$$ (1)

where *V*_wash_ is the volume of wash-off suspension (3 ml), *V*_droplet_ is the volume of the droplets in the freezing assay experiment (1 μl), and *V*_air_ is the volume of sampled air at standard temperature and pressure.

For each filter, experiments were repeated three times and combined by binning the data into 1°C temperature intervals and finding a mean number of freezing events in each bin and then the mean fraction frozen. The error bars represent the SDs of these repeat runs. In addition, the influence of background INP activity was removed by background subtraction similar to the method described by Sanchez-Marroquin *et al.* (*21*). Briefly, for each handling blank and sample, the differential freezing spectra [*k*(*T*)] were calculated. Then, the mean and SD of *k*(*T*) for all the handling blanks were calculated and taken to represent our background activity. This background *k*(*T*) value was subtracted from the mean *k*(*T*) of each sample, and the SDs were combined in quadrature to represent the total error. After background subtraction, *k*(*T*) was converted to cumulative INP spectra, *K*(*T*), from which *N*_INP_ was calculated. For data points falling in the background, this subtraction results in a *k*(*T*) value of zero and no increase in *K*(*T*) or *N*_INP_ at this temperature interval in the cumulative space; however, these points would still have an upper error bar above zero. Hence, the measured INP activity at that temperature interval is consistent with zero, but the top of the error bar represents a possible upper limit.

### Heat tests

We performed a heat test according to the protocol defined by Daily *et al.* (*36*). Suspensions were prepared as previously outlined, and then, a 1-ml aliquot of liquid containing the sample was separated. This was placed in a 15-ml centrifuge tube and then heated in a vessel of boiling water for 30 min. The liquid was allowed to cool and then tested using a standard μL-NIPI droplet freezing assay.

### Gravimetric analysis and ice-active site density calculation

To make comparisons of ice-nucleating activity across different samples, the surface area of material per droplet can be used to normalize the data and give a value of the number of active sites per unit surface area, *n*_*s*_(*T*)

$$n_{s}(T)=-\frac{\ln[1-f_{\mathrm{ice}}(T)]}{A_{s}}$$ (2)

where *A*_*s*_ is the total surface area of particles per droplet. This was estimated for each impactor size bin using the mass of sampled aerosol, the average specific surface area of particles in each bin, and the known droplet and suspension volumes (1 µl and 3 ml, respectively).

The mass of aerosol sampled in each size bin was determined gravimetrically. Before preparing the suspensions as described above, each filter was weighed using a microbalance (Sartorius Cubis High-Capacity Micro Balance; Sartorius Ltd.). After washing, the filter was removed from the suspension and dried in an oven at 50°C for approximately 1 hour and weighed again. The difference in mass before and after washing was then taken as the sampled aerosol mass for that size bin. Measurements of such low masses can have a high uncertainty, so each filter was weighed five times and the mean and SD were calculated. In addition, filters were placed under an antistatic fan before every measurement to minimize errors associated with a buildup of static electricity. To ensure that the process did not alter the mass of the filters themselves, 20 new filters that had not been exposed to aerosol were analyzed using the same process, and the uncertainty was found to be ±5μg, which was taken to be the limit of detection for this method. Last, droplet freezing experiments using the wash-off suspensions from the blank filters were used to confirm that there was no increase in background INP activity; therefore, we can assume that the process does not introduce contamination.

The average specific surface area of particles in each size bin was estimated using Eq. 3

$$\mathrm{SSA}=\frac{6}{\rho d}$$ (3)

where ρ is the density of the particle and *d* is the diameter of the particle. A value of 2.65 g cm^−3^ was used for ρ, which represents mineral dust, and *d* was approximated by using the diameter at the middle of each size bin. For stage A, which does not have a defined upper size boundary, we used a diameter of 4 μm because we were unlikely to collect particles >6 μm because of particle losses as a result of high wind speeds, as outlined above.

### Particle dispersion and dust concentration modeling

The transport dust from the Copper River was modeled using FLEXPART (*13*, *51*). FLEXPART is a Lagrangian particle dispersion model used to simulate the transport of air parcels by mean flow and processes such as turbulent and diffusive transport, turbulence, and convection (*51*). We used FLEXPART to run 10-day forward trajectories of particles released over a 4-day period; the results presented here correspond to FLEXPART runs starting at 00:00 on 14 October 2019. Particles were released between 0 and 10 m above the surface in a 300-km^2^ region covering the lower reaches of the Copper River Delta and encompassing our sampling sites. Model runs were driven by ERA5 meteorological reanalysis data from the European Centre for Medium Range Weather Forecast (*46*) with a 3-hour temporal resolution, 0.28° × 0.28° (30 km) horizontal resolution and 137 vertical levels. We used an aerosol tracer with characteristics tuned to represent mineral dust and particularly to accurately represent wet and dry aerosol removal processes. Wet deposition in FLEXPART is partitioned into below-cloud and in-cloud processes, taking into account scavenging by rain and snow, as well as the efficiency of particles to act as cloud condensation nuclei or INPs. Scavenging coefficients were chosen on the basis of findings of a multiyear study of mineral dust deposition using FLEXPART (*15*). Particle sizes in FLEXPART are defined by a log-normal distribution around a mean particle diameter, where the user can specify the mean and sigma values. We tested different particle sizes, corresponding to the size bins of the impactor stages; however, the results presented here correspond to a mean particle diameter of 1 μm because accumulation mode dust particles dominate the number of dust INPs because of their long lifetime and high concentration. A comparison of modeled INP concentration with different mean particle sizes is shown in fig. S3. When running forward trajectories, the total mass emitted can be specified at the start of each model run. Estimates of the total dust mass from a snapshot of a dust plume during a large event in 2006 ranged from 9 to 26 kt; this event continued intermittently for 18 days, and hence, it was expected that the total mass emitted over the full period was considerably higher (30 to 80 kt) (*31*). Comparing satellite imagery from this event to our sampling period suggests that the total emissions during the event that we sampled are likely to be considerably less. To capture the possible range of emissions from dust events and the resulting atmospheric INP concentrations, we repeated FLEXPART runs with total masses ranging from 1 to 80 kt (fig. S4).

### Modeled INP concentrations

From our calculations of *n*_*s*_, we developed parametrizations of *n*_*s*_(*T*), representing dust from the Copper River. For each of the four impactor stages, we calculated a mean *n*_*s*_(*T*) curve. We then fitted a second-order polynomial to the logarithm of these mean values to yield four different parametrizations based on particle size. Combining *n*_*s*_(*T*) with dust concentration and temperature leads to atmospheric INP concentrations, *N*_INP_(*T*)

$$N_{\mathrm{INP}}(T)=N_{\mathrm{dust}}\left\{1-\exp[-n_{s}(T)s]\right\}$$ (4)

*n*_*s*_(*T*) is the ice-active site density at temperature *T*; *s* is the surface area of an individual particle, and *N*_dust_ is the dust number concentration. In this case, *N*_dust_ can be determined from FLEXPART model results, and *T* was set to the ambient atmospheric temperature (*T*_amb_) from ERA5 reanalysis data to determine *N*_INP_(*T*) or the number of particles that might activate to ice at *T* and within a cloud droplet. The results from model runs shown here represent a particle size of 1 μm; hence, we used the *n*_*s*_ parametrization for stage B, which corresponds to 1 to 2.5 μm, and calculations of *s* are based on a particle diameter of 1 μm. In addition, dust mass concentrations from FLEXPART were converted to *N*_dust_ based on a particle diameter of 1 μm and a density of 2.65 g cm^−3^.

## References

[1] U. Lohmann, J. Feichter, Global indirect aerosol effects: A review. Atmos. Chem. Phys. 5, 715–737 (2005).

[2] P. Ceppi, F. Brient, M. D. Zelinka, D. L. Hartmann, Cloud feedback mechanisms and their representation in global climate models. WIREs Climate Change 8, e465 (2017).

[3] I. Tan, T. Storelvmo, M. D. Zelinka, Observational constraints on mixed-phase clouds imply higher climate sensitivity. Science 352, 224–227 (2016). 27124459 10.1126/science.aad5300

[4] J. Vergara-Temprado, A. K. Miltenberger, K. Furtado, D. P. Grosvenor, B. J. Shipway, A. A. Hill, J. M. Wilkinson, P. R. Field, B. J. Murray, K. S. Carslaw, Strong control of Southern Ocean cloud reflectivity by ice-nucleating particles. Proc. Natl. Acad. Sci. U.S.A. 115, 2687–2692 (2018). 29490918 10.1073/pnas.1721627115 PMC5856555

[5] B. J. Murray, K. S. Carslaw, P. R. Field, Opinion: Cloud-phase climate feedback and the importance of ice-nucleating particles. Atmos. Chem. Phys. 21, 665–679 (2021).

[6] M. D. Shupe, Clouds at arctic atmospheric observatories. Part II: Thermodynamic phase characteristics. J. Appl. Meteorol. Climatol. 50, 645–661 (2011).

[7] P. J. DeMott, K. Sassen, M. R. Poellot, D. Baumgardner, D. C. Rogers, S. D. Brooks, A. J. Prenni, S. M. Kreidenweis, African dust aerosols as atmospheric ice nuclei. Geophys. Res. Lett. 30, 1732 (2003).

[8] K. Sassen, P. J. DeMott, J. M. Prospero, M. R. Poellot, Saharan dust storms and indirect aerosol effects on clouds: CRYSTAL-FACE results. Geophys. Res. Lett. 30, 1633 (2003).

[9] J. D. Atkinson, B. J. Murray, M. T. Woodhouse, T. F. Whale, K. J. Baustian, K. S. Carslaw, S. Dobbie, D. O’Sullivan, T. L. Malkin, D. O’Sullivan, T. L. Malkin, The importance of feldspar for ice nucleation by mineral dust in mixed-phase clouds. Nature 498, 355–358 (2013). 23760484 10.1038/nature12278

[10] Y. Boose, B. Sierau, M. I. García, S. Rodríguez, A. Alastuey, C. Linke, M. Schnaiter, P. Kupiszewski, Z. A. Kanji, U. Lohmann, Ice nucleating particles in the Saharan Air Layer. Atmos. Chem. Phys. 16, 9067–9087 (2016).

[11] T. Storelvmo, I. Tan, A. V. Korolev, Cloud phase changes induced by CO_2_ warming—A powerful yet poorly constrained cloud-climate feedback. Curr. Clim. Change Rep. 1, 288–296 (2015).

[12] J. Vergara-Temprado, B. J. Murray, T. W. Wilson, D. O’Sullivan, J. Browse, K. J. Pringle, K. Ardon-Dryer, A. K. Bertram, S. M. Burrows, D. Ceburnis, P. J. Demott, R. H. Mason, C. D. O’Dowd, M. Rinaldi, K. S. Carslaw, J. Browse, K. J. Pringle, K. Ardon-Dryer, A. K. Bertram, S. M. Burrows, D. Ceburnis, P. J. Demott, R. H. Mason, M. Rinaldi, K. S. Carslaw, Contribution of feldspar and marine organic aerosols to global ice nucleating particle concentrations. Atmos. Chem. Phys. 17, 3637–3658 (2017).

[13] A. Stohl, Characteristics of atmospheric transport into the Arctic troposphere. J. Geophys. Res. 111, D11306 (2006).

[14] L. B. Hande, C. Engler, C. Hoose, I. Tegen, Seasonal variability of Saharan desert dust and ice nucleating particles over Europe. Atmos. Chem. Phys. 15, 4389–4397 (2015).

[15] C. D. Groot Zwaaftink, H. Grythe, H. Skov, A. Stohl, Substantial contribution of northern high-latitude sources to mineral dust in the Arctic. J. Geophys. Res. 121, 13,678–13,697 (2016). 10.1002/2016JD025482 PMC6686616 31423407

[16] Y. Shi, X. Liu, M. Wu, X. Zhao, Z. Ke, H. Brown, Relative importance of high-latitude local and long-range-transported dust for Arctic ice-nucleating particles and impacts on Arctic mixed-phase clouds. Atmos. Chem. Phys. 22, 2909–2935 (2022).

[17] J. E. Bullard, B. Matthew, B. Tom, C. John, D. Eleanor, G. Diego, G. Santiago, G. Gudrun, H. Richard, M. Robert, M.-N. Cheryl, M. Tom, S. Helena, T. Thorsteinsson, High latitude dust in the Earth system. Rev. Geophys. 54, 447–485 (2016).

[18] O. Meinander, P. Dagsson-Waldhauserova, P. Amosov, E. Aseyeva, C. Atkins, A. Baklanov, C. Baldo, S. L. Barr, B. Barzycka, L. G. Benning, B. Cvetkovic, P. Enchilik, D. Frolov, S. Gassó, K. Kandler, N. Kasimov, J. Kavan, J. King, T. Koroleva, V. Krupskaya, M. Kulmala, M. Kusiak, H. K. Lappalainen, M. Laska, J. Lasne, M. Lewandowski, B. Luks, J. B. McQuaid, B. Moroni, B. Murray, O. Möhler, A. Nawrot, S. Nickovic, N. T. O’Neill, G. Pejanovic, O. Popovicheva, K. Ranjbar, M. Romanias, O. Samonova, A. Sanchez-Marroquin, K. Schepanski, I. Semenkov, A. Sharapova, E. Shevnina, Z. Shi, M. Sofiev, F. Thevenet, T. Thorsteinsson, M. Timofeev, N. S. Umo, A. Uppstu, D. Urupina, G. Varga, T. Werner, O. Arnalds, A. Vimic, Newly identified climatically and environmentally significant high-latitude dust sources. Atmos. Chem. Phys. 22, 11889–11930 (2022).

[19] C. D. Groot Zwaaftink, Ó. Arnalds, P. Dagsson-Waldhauserova, S. Eckhardt, J. M. Prospero, A. Stohl, C. G. Zwaaftink, Temporal and spatial variability of Icelandic dust emissions and atmospheric transport. Atmos. Chem. Phys. 17, 10865–10878 (2017).

[20] Y. Tobo, K. Adachi, P. J. Demott, T. C. J. Hill, D. S. Hamilton, N. M. Mahowald, N. Nagatsuka, S. Ohata, J. Uetake, Y. Kondo, M. Koike, Glacially sourced dust as a potentially significant source of ice nucleating particles. Nat.Geosci. 12, 253–258 (2019).

[21] A. Sanchez-Marroquin, O. Arnalds, K. J. Baustian-Dorsi, J. Browse, P. Dagsson-Waldhauserova, A. D. Harrison, E. C. Maters, K. J. Pringle, J. Vergara-Temprado, I. T. Burke, J. B. McQuaid, K. S. Carslaw, B. J. Murray, Iceland is an episodic source of atmospheric ice-nucleating particles relevant for mixed-phase clouds. Sci. Adv. 6, eaba8137 (2020). 32637618 10.1126/sciadv.aba8137 PMC7314534

[22] Y. Xi, C. Xu, A. Downey, R. Stevens, J. O. Bachelder, J. King, P. L. Hayes, A. K. Bertram, Ice nucleating properties of airborne dust from an actively retreating glacier in Yukon, Canada. Environ. Sci. 2, 714–726 (2022).

[23] C. Baldo, P. Formenti, S. Nowak, S. Chevaillier, M. Cazaunau, E. Pangui, C. Di Biagio, J.-F. Doussin, K. Ignatyev, P. Dagsson-Waldhauserova, O. Arnalds, A. R. MacKenzie, Z. Shi, Distinct chemical and mineralogical composition of Icelandic dust compared to northern African and Asian dust. Atmos. Chem. Phys. 20, 13521–13539 (2020).

[24] Z. Brasseur, D. Castarède, E. S. Thomson, M. P. Adams, S. Drossaart van Dusseldorp, P. Heikkilä, K. Korhonen, J. Lampilahti, M. Paramonov, J. Schneider, F. Vogel, Y. Wu, J. P. D. Abbatt, N. S. Atanasova, D. H. Bamford, B. Bertozzi, M. Boyer, D. Brus, M. I. Daily, R. Fösig, E. Gute, A. D. Harrison, P. Hietala, K. Höhler, Z. A. Kanji, J. Keskinen, L. Lacher, M. Lampimäki, J. Levula, A. Manninen, J. Nadolny, M. Peltola, G. C. E. Porter, P. Poutanen, U. Proske, T. Schorr, N. Silas Umo, J. Stenszky, A. Virtanen, D. Moisseev, M. Kulmala, B. J. Murray, T. Petäjä, O. Möhler, J. Duplissy, Measurement report: Introduction to the HyICE-2018 campaign for measurements of ice-nucleating particles and instrument inter-comparison in the Hyytiälä boreal forest. Atmos. Chem. Phys. 22, 5117–5145 (2022).

[25] F. Conen, S. Eckhardt, H. Gundersen, A. Stohl, K. E. Yttri, Rainfall drives atmospheric ice-nucleating particles in the coastal climate of southern Norway. Atmos. Chem. Phys. 17, 11065–11073 (2017).

[26] J. Crusius, A. W. Schroth, J. A. Resing, J. Cullen, R. W. Campbell, Seasonal and spatial variabilities in northern Gulf of Alaska surface water iron concentrations driven by shelf sediment resuspension, glacial meltwater, a Yakutat eddy, and dust. Glob. Biogeochem. Cycles 31, 942–960 (2017).

[27] R. S. Tarr, L. Martin, Glacial deposits of the continental type in Alaska. J. Geol. 21, 289–300 (1913).

[28] J. S. Kargel, M. J. Beedle, A. B. Bush, F. Carreño, E. Castellanos, U. K. Haritashya, G. J. Leonard, J. Lillo, I. Lopez, M. Pleasants, E. Pollock, D. F. Wolfe. in *Global Land Ice Measurements from Space*, J. S. Kargel, G. J. Leonard, M. P. Bishop, A. Kääb, B. H. Raup, eds. (Springer Praxis Books, Springer, 2014), pp. 297–332.

[29] T. P. Brabets, *Geomorphology of the Lower Copper River, Alaska, no. 1581*, in U.S. Geological Survey Professional Paper (U.S. G.P.O., 1997).

[30] J. M. Jaeger, C. A. Nittrouer, N. D. Scott, J. D. Milliman, Sediment accumulation along a glacially impacted mountainous coastline: Northeast Gulf of Alaska. Basin Res. 10, 155–173 (1998).

[31] J. Crusius, A. W. Schroth, S. Gassó, C. M. Moy, R. C. Levy, M. Gatica, Glacial flour dust storms in the Gulf of Alaska: Hydrologic and meteorological controls and their importance as a source of bioavailable iron. Geophys. Res. Lett. 38, 10.1029/2010GL046573 (2011).

[32] A. W. Schroth, J. Crusius, S. Gassó, C. M. Moy, N. J. Buck, J. A. Resing, R. W. Campbell, Atmospheric deposition of glacial iron in the Gulf of Alaska impacted by the position of the Aleutian Low. Geophys. Res. Lett. 44, 5053–5061 (2017). 32636573 10.1002/2017gl073565 PMC7340097

[33] R. H. Mason, M. Si, J. Li, C. Chou, R. Dickie, D. Toom-Sauntry, C. Pöhlker, J. D. Yakobi-Hancock, L. A. Ladino, K. Jones, W. R. Leaitch, C. L. Schiller, J. P. D. Abbatt, J. A. Huffman, A. K. Bertram, Ice nucleating particles at a coastal marine boundary layer site: Correlations with aerosol type and meteorological conditions. Atmos. Chem. Phys. 15, 12547–12566 (2015).

[34] J. M. Creamean, R. M. Kirpes, K. A. Pratt, N. J. Spada, M. Maahn, G. De Boer, R. C. Schnell, S. China, Marine and terrestrial influences on ice nucleating particles during continuous springtime measurements in an Arctic oilfield location. Atmos. Chem. Phys. 18, 18023–18042 (2018).

[35] M. Si, E. Evoy, J. Yun, Y. Xi, S. J. Hanna, A. Chivulescu, K. Rawlings, D. Veber, A. Platt, D. Kunkel, P. Hoor, S. Sharma, W. Richard Leaitch, A. K. Bertram, W. R. Leaitch, A. K. Bertram, Concentrations, composition, and sources of ice-nucleating particles in the Canadian High Arctic during Spring 2016. Atmos. Chem. Phys. 19, 3007–3024 (2019).

[36] M. I. Daily, M. D. Tarn, T. F. Whale, B. J. Murray, An evaluation of the heat test for the ice-nucleating ability of minerals and biological material. Atmos. Meas. Tech. 15, 2635–2665 (2022).

[37] N. Reicher, L. Segev, Y. Rudich, The WeIzmann Supercooled Droplets Observation on a Microarray (WISDOM) and application for ambient dust. Atmos. Meas. Tech. 11, 233–248 (2018).

[38] G. C. E. Porter, M. P. Adams, I. M. Brooks, L. Ickes, L. Karlsson, C. Leck, M. E. Salter, J. Schmale, K. Siegel, S. N. F. Sikora, M. D. Tarn, J. Vüllers, H. Wernli, P. Zieger, J. Zinke, B. J. Murray, Highly active ice-nucleating particles at the Summer North Pole. J. Geophys. Res. Atmos. 127, e2021JD036059 (2022). 10.1029/2021JD036059 PMC9285974 35865411

[39] A. D. Harrison, K. Lever, A. Sanchez-Marroquin, M. A. Holden, T. F. Whale, M. D. Tarn, J. B. McQuaid, B. J. Murray, The ice-nucleating ability of quartz immersed in water and its atmospheric importance compared to K-feldspar. Atmos. Chem. Phys. 19, 11343–11361 (2019).

[40] A. D. Harrison, D. O’Sullivan, M. P. Adams, G. C. E. Porter, E. Blades, C. Brathwaite, R. Chewitt-Lucas, C. Gaston, R. Hawker, O. O. Krüger, L. Neve, M. L. Pöhlker, C. Pöhlker, U. Pöschl, A. Sanchez-Marroquin, A. Sealy, P. Sealy, M. D. Tarn, S. Whitehall, J. B. McQuaid, K. S. Carslaw, J. M. Prospero, B. J. Murray, The ice-nucleating activity of African mineral dust in the Caribbean boundary layer. Atmos. Chem. Phys. 22, 9663–9680 (2022).

[41] H. C. Price, K. J. Baustian, J. B. McQuaid, A. Blyth, K. N. Bower, T. Choularton, R. J. Cotton, Z. Cui, P. R. Field, M. Gallagher, R. Hawker, A. Merrington, A. Miltenberger, R. R. Neely, S. T. Parker, P. D. Rosenberg, J. W. Taylor, J. Trembath, J. Vergara-Temprado, T. F. Whale, T. W. Wilson, G. Young, B. J. Murray, Atmospheric ice-nucleating particles in the dusty tropical Atlantic. J. Geophys. Res. Atmos. 123, 2175–2193 (2018).

[42] M. Paramonov, R. O. David, R. Kretzschmar, Z. A. Kanji, A laboratory investigation of the ice nucleation efficiency of three types of mineral and soil dust. Atmos. Chem. Phys. 18, 16515–16536 (2018).

[43] T. F. Whale, M. A. Holden, T. W. Wilson, D. O’Sullivan, B. J. Murray, The enhancement and suppression of immersion mode heterogeneous ice-nucleation by solutes. Chem. Sci. 9, 4142–4151 (2018). 29780544 10.1039/c7sc05421a PMC5941198

[44] D. O’Sullivan, B. J. Murray, J. F. Ross, T. F. Whale, H. C. Price, J. D. Atkinson, N. S. Umo, M. E. Webb, The relevance of nanoscale biological fragments for ice nucleation in clouds. Sci. Rep. 5, 8082 (2015). 25626414 10.1038/srep08082 PMC4308702

[45] J. Crusius, Dissolved Fe supply to the Central Gulf of Alaska is inferred to be derived from Alaskan glacial dust that is not resolved by dust transport models. J. Geophys. Res. Biogeosci. 126, e2021JG006323 (2021).

[46] H. Hersbach, B. Bell, P. Berrisford, S. Hirahara, A. Horányi, J. Muñoz-Sabater, J. Nicolas, C. Peubey, R. Radu, D. Schepers, A. Simmons, C. Soci, S. Abdalla, X. Abellan, G. Balsamo, P. Bechtold, G. Biavati, J. Bidlot, M. Bonavita, G. Chiara, P. Dahlgren, D. Dee, M. Diamantakis, R. Dragani, J. Flemming, R. Forbes, M. Fuentes, A. Geer, L. Haimberger, S. Healy, R. J. Hogan, E. Hólm, M. Janisková, S. Keeley, P. Laloyaux, P. Lopez, C. Lupu, G. Radnoti, P. Rosnay, I. Rozum, F. Vamborg, S. Villaume, J.-N. Thépaut, *ERA5 Hourly Data on Pressure Levels from 1959 to Present* (Copernicus Climate Change Service Climate Data Store, 2020); 10.24381/cds.bd0915c6.

[47] K. Kawai, H. Matsui, Y. Tobo, Dominant role of arctic dust with high ice nucleating ability in the Arctic lower troposphere. Geophys. Res. Lett. 50, e2022GL102470 (2023).

[48] G. C. Porter, S. N. Sikora, M. P. Adams, U. Proske, A. D. Harrison, M. D. Tarn, I. M. Brooks, B. J. Murray, Resolving the size of ice-nucleating particles with a balloon deployable aerosol sampler: The SHARK. Atmos. Meas. Tech. 13, 2905–2921 (2020).

[49] S.-L. von der Weiden, F. Drewnick, S. Borrmann, Particle Loss Calculator—A new software tool for the assessment of the performance of aerosol inlet systems. Atmos. Meas. Tech. 2, 479–494 (2009).

[50] T. F. Whale, B. J. Murray, D. Sullivan, T. W. Wilson, N. S. Umo, K. J. Baustian, J. D. Atkinson, D. A. Workneh, G. J. Morris, A technique for quantifying heterogeneous ice nucleation in microlitre supercooled water droplets. Atmos. Meas. Tech. 8, 2437–2447 (2015).

[51] I. Pisso, E. Sollum, H. Grythe, N. I. Kristiansen, M. Cassiani, S. Eckhardt, D. Arnold, D. Morton, R. L. Thompson, C. D. Groot Zwaaftink, N. Evangeliou, H. Sodemann, L. Haimberger, S. Henne, D. Brunner, J. F. Burkhart, A. Fouilloux, J. Brioude, A. Philipp, P. Seibert, A. Stohl, The Lagrangian particle dispersion model FLEXPART version 10.4. Geosci. Model Dev. 12, 4955–4997 (2019).

[52] A. Sanchez-Marroquin, D. H. P. Hedges, M. Hiscock, S. T. Parker, P. D. Rosenberg, J. Trembath, R. Walshaw, I. T. Burke, J. B. McQuaid, B. J. Murray, Characterisation of the filter inlet system on the BAE-146 research aircraft and its use for size resolved aerosol composition measurements. Atmos. Meas. Tech. 12, 5741–5763 (2019).

