# Supplementary material for: Southern Alaska as a source of atmospheric mineral dust and ice-nucleating particles
Source: Sci Adv. 2023 Aug 16;9(33):eadg3708. doi: 10.1126/sciadv.adg3708 (PMC10431707; doi:10.1126/sciadv.adg3708)
Supplement: Supplementary file 1 — Figs. S1 to S4 Table S1 [file sciadv.adg3708_sm.pdf]

Science Advances
AAAS

Supplementary Materials for

Southern Alaska as a source of atmospheric mineral dust and ice-nucleating particles

Sarah L. Barr *et al.*

Corresponding author: Sarah L. Barr, eeslb@leeds.ac.uk

Sci. Adv. **9**, eadg3708 (2023)
DOI: 10.1126/sciadv.adg3708

This PDF file includes:

Figs. S1 to S4
Table S1

Fig. S1.

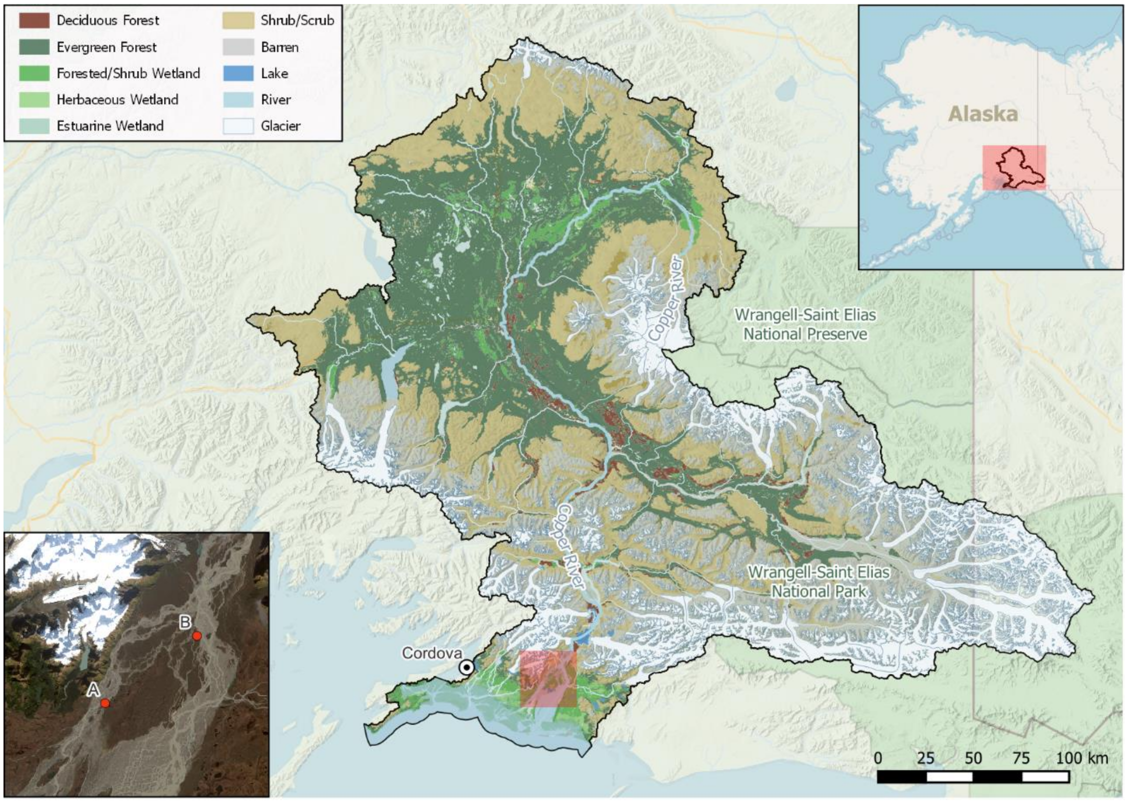

Map of the Copper River watershed showing vegetation types. Land cover data from U.S. Geological Survey National Land Cover Database (NLCD), geospatial data such as waterways, boundaries, National Parks from State of Alaska Open Data Geoportal, background map from MapTiler and OpenStreetMap, lower left inset derived from Landsat 8 Collection 2 Tier 1 calibrated top-of-atmosphere (TOA) reflectance

Fig. S2.

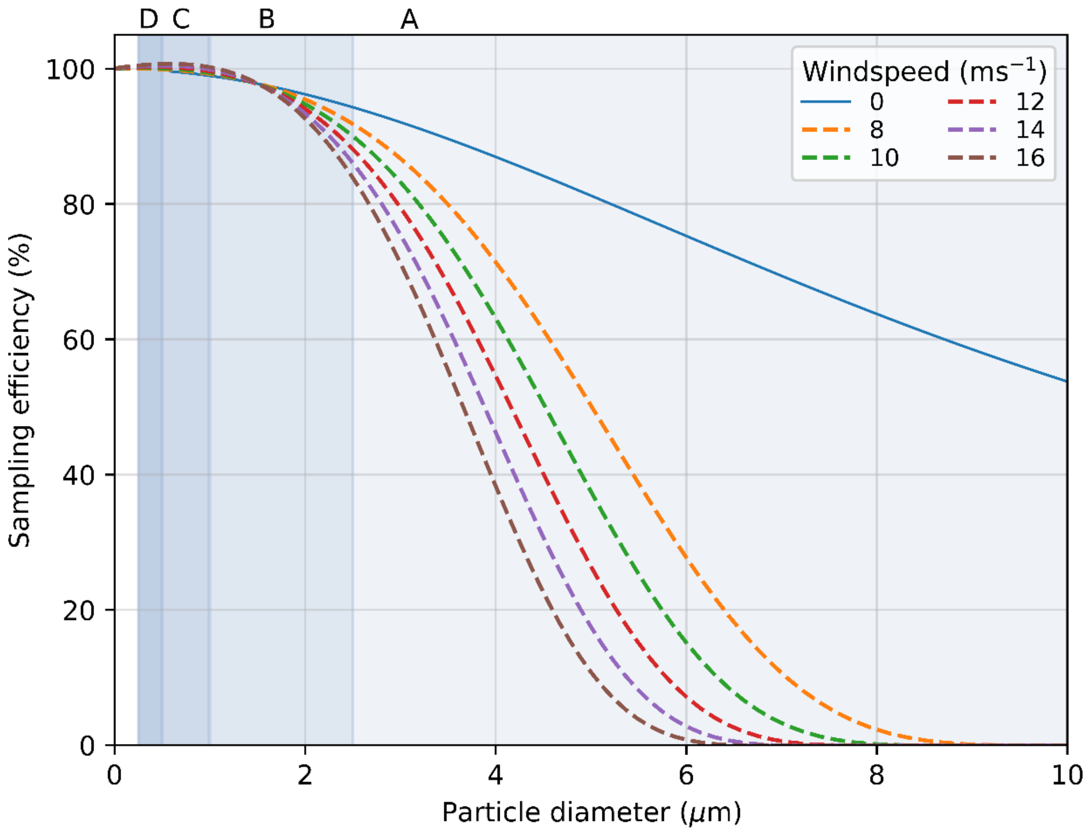

Sampling efficiency of the impactor at different wind speeds. Shaded regions represent the different size bins of the impactor (A: > 2.5 µm, B: 1-2. µm, C: 0.5-1 µm, D: 0.25-0.5 µm)

Fig. S3.

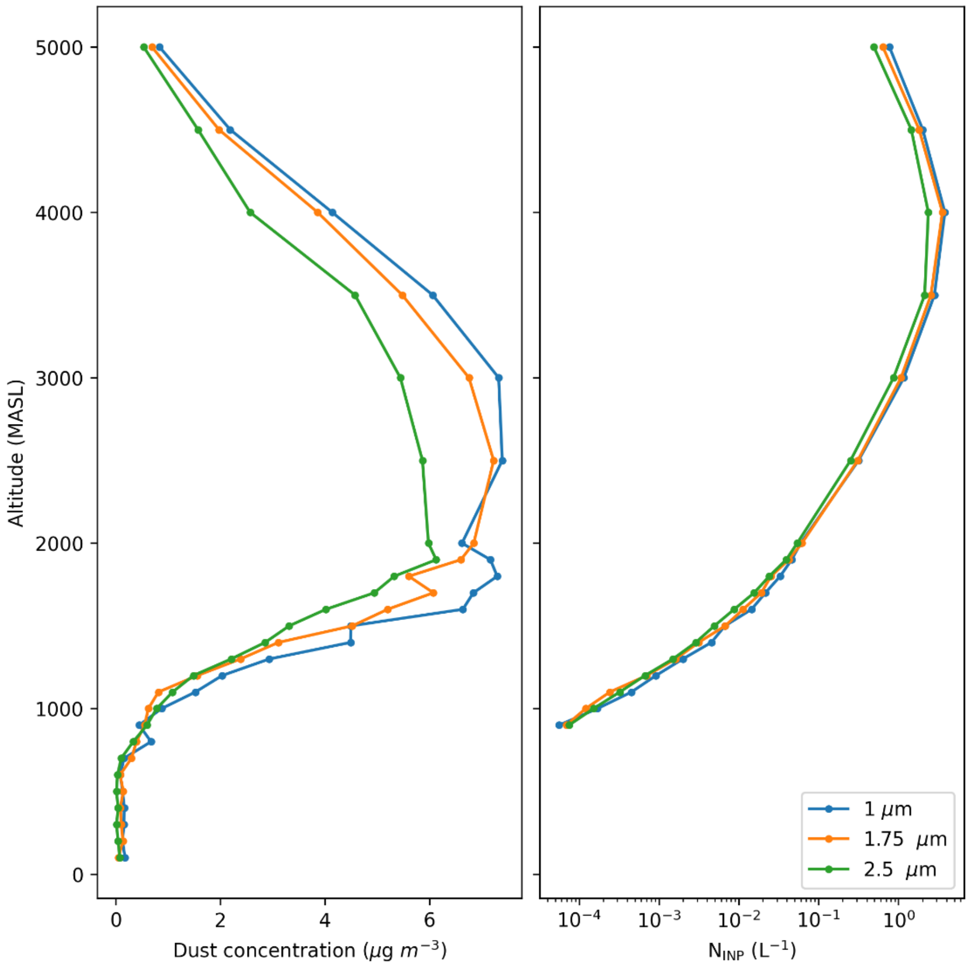

Comparison of modelled dust concentration and N_{INP} with varying mean particle size in 10 day FLEXPART simulations starting on 14th October 2019. All model parameters are the same as for runs presented in the main body of the paper, including a total emitted mass of 15 kt.

Fig. S4.

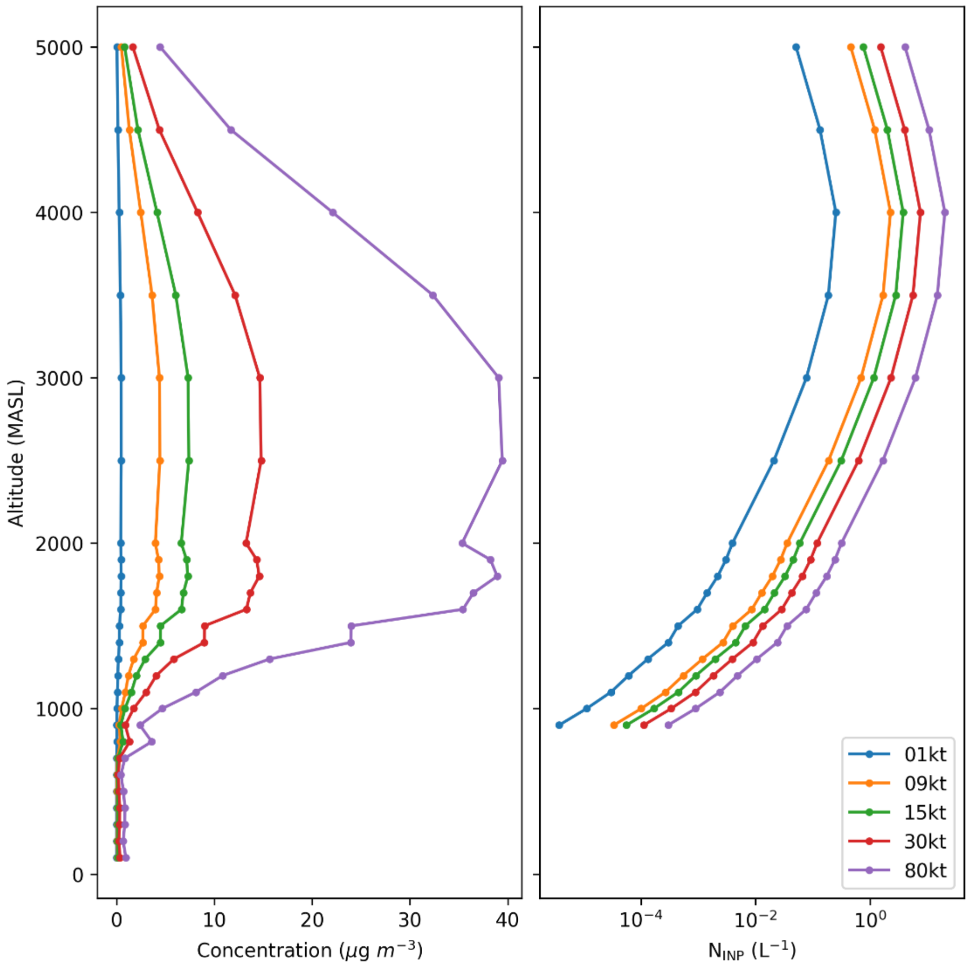

Comparison of modelled dust concentration and N\textsubscript{INP} with varying initial dust emissions mass in 10 day FLEXPART simulations starting on 14th October 2019. All model parameters are the same as model runs presented in the main body of the paper, including a mean particle size of 1 μm

Table S1.

**Albite**	57.61%
**Quartz**	15.41%
**Microcline**	8.94%
**Calcite**	7.99%
**Muscovite**	5.86%
**Chlorite**	2.51%
**Riebeckite**	1.35%
**Montmorillonite**	0.33%

Results of X-Ray diffraction on 45 μm surface samples showing the percentage of each mineral in the sample determined using Total Pattern Analysis Solutions (TOPAS) analysis of Rietveld refinement of powder X-ray diffraction (XRD) patterns
